# Integrative Bioinformatics Analysis Reveals Key Regulatory Genes and Therapeutic Targets in Ulcerative Colitis Pathogenesis

**DOI:** 10.3390/genes16111296

**Published:** 2025-11-01

**Authors:** Sheikh Atikur Rahman, Mst. Tania Khatun, Mahendra Singh, Viplov Kumar Biswas, Forkanul Hoque, Nurun Nesa Zaman, Anzana Parvin, Mohammad Khaja Mafij Uddin, Md. Mominul Islam Sheikh, Most Morium Begum, Rakesh Arya, Hossain Md. Faruquee

**Affiliations:** 1 Department of Biotechnology and Genetic Engineering, Faculty of Biological Sciences, Islamic University, Kushtia 7003, Bangladesh; skatikurrahman347@gmail.com (S.A.R.); nesazaman1234@gmail.com (N.N.Z.); anzana@btge.iu.ac.bd (A.P.); 2 Department of Pharmacy, Faculty of Biological Sciences, Islamic University, Kushtia 7003, Bangladesh; taniakhatun9090@gmail.com (M.T.K.); forkanshah22@gmail.com (F.H.); 3 Department of Biotechnology, Yeungnam University, Gyeongsan 38541, Republic of Korea; m.singh2685@gmail.com; 4 Department of Pathology and Laboratory Medicine, Emory University School of Medicine, Whitehead Biomedical Research, 615 Michael Street, Atlanta, GA 30322, USA; viplov.kumar.biswas@emory.edu; 5 Infectious Diseases Division, International Centre for Diarrhoeal Disease Research, Dhaka 1212, Bangladesh; kmuddin@icddrb.org; 6 Nano Project Group, Research & Development Institute, Moorim P&P Co., Ltd., Ulju-gun, Ulsan 45011, Republic of Korea; momin.gnu@gmail.com; 7 Department of Agriculture, Rabindra Maitree University, Kushtia 7000, Bangladesh; moriumru@gmail.com

**Keywords:** ulcerative colitis, biomarkers, hub genes, bioinformatics, immune response, network analysis, molecular docking, drug candidates, diagnosis

## Abstract

Background: Ulcerative colitis (UC), a chronic and relapsing form of inflammatory bowel disease (IBD), arises from a multifactorial interplay of genetic predisposition, immune dysregulation, and environmental triggers. Despite advances in understanding UC pathogenesis, the identification of reliable biomarkers and key regulatory genes remains essential for unraveling disease mechanisms. Such insights are crucial for improving diagnostic precision and developing personalized therapeutic strategies. Methods: In this study, gene expression profiles from publicly available microarray and RNA-sequencing datasets were systematically analyzed using advanced bioinformatics tools. Differentially expressed genes (DEGs) were identified through statistical comparisons, and functional enrichment analyses were performed to explore their biological relevance. A total of 141 overlapping DEGs were extracted from three GEO datasets, and 20 key DEGs were further prioritized via protein–protein interaction (PPI) network construction. Hub genes, relevant signaling pathways, associated transcription factors (TFs), and microRNAs (miRNAs) linked to disease progression were identified. Potential therapeutic compounds were also predicted through computational drug–gene interaction analysis. Results: The analysis revealed a panel of novel biomarkers-TLR2, IFNG, CD163, CXCL9, CCL4, PRF1, TLR8, ARG1, LILRB2, FPR2, and PPARG-that function as key hub genes implicated in ulcerative colitis (UC) pathogenesis. These genes were associated with critical biological processes including signal transduction, inflammatory and immune responses, proteolysis, lipid transport, and cholesterol/triglyceride homeostasis. Furthermore, transcription factors (FOXC1, GABPA, GATA2, SUPT5H) and microRNAs (hsa-miR-34a-5p, hsa-miR-335-5p, hsa-miR-24-3p, hsa-miR-23a-5p, hsa-miR-26a-5p) revealed key regulatory networks influencing post-transcriptional gene regulation. Molecular docking analysis predicted Apremilast and Golotimod as promising therapeutic candidates for UC intervention. Conclusions: In conclusion, this study enhances our understanding of ulcerative colitis pathogenesis by identifying key biomarkers and therapeutic targets, paving the way for future advancements in personalized diagnosis and treatment strategies.

## 1. Introduction

Ulcerative colitis (UC) is a long-term inflammatory disorder of the large intestine, primarily impacting the colon and rectum [1]. It typically originates in the rectum and extends proximally through the colon, either partially or completely [2]. UC is a persistent, relapsing condition of unknown origin and has been increasingly reported worldwide [3]. Its complex pathogenesis involves a multifactorial interplay of environmental, microbial, and genetic factors that disrupt mucosal homeostasis and trigger inappropriate immune responses [4]. UC substantially impairs quality of life, often resulting in complications such as diarrhea, bloody mucous-laden stools, tenesmus, fecal urgency, incontinence, fatigue, and abdominal pain [5]. It is also a significant precursor to colorectal cancer (CRC), with risk increasing proportionally to the duration of chronic intestinal inflammation [6,7]. Some studies suggest an exponential rise in annual CRC risk. UC accounts for approximately 15% of IBD-related mortality, with an incidence rate 1.5 to 2.4 times higher than in the general population [8,9]. Over time, the global incidence of UC has risen significantly, and by 2035, UC is projected to affect over two million individuals in Asia [10].

Although multiple therapeutic options exist, up to 15% of patients exhibit treatment resistance or develop colitis with dysplastic changes requiring surgical intervention [11]. In cases of acute severe ulcerative colitis (ASUC), oral or intravenous drug delivery may be compromised by systemic side effects and poor site-specific drug availability, which may restrict therapeutic effectiveness [12,13]. Differentiating UC from other conditions can be difficult, as diagnosis requires a thorough assessment of clinical symptoms, endoscopic findings, and histological features. Due to its uncertain etiology and lack of precise diagnostic markers, identifying predictive biomarkers remains critical for timely diagnosis and targeted therapy.

Currently, no approved biomarkers or commercial assays are available for early UC detection in clinical settings. However, bioinformatics approaches have been extensively used to explore disease-related molecular mechanisms and uncover novel biomarkers. Recent advances, including RNA sequencing and mucosal gene expression profiling, have yielded several candidate genes. Notable among these are Cadherin 11, hepatocyte nuclear factor 4 alpha (HNF4α), intercellular adhesion molecule 1 (ICAM1), and ring finger protein 186 (RNF186) [14]. These genes are linked to essential cellular functions, including immune modulation, epithelial repair, barrier integrity, microbial defense, autophagy, cell proliferation, and apoptosis [15,16]. Still, the specificity and sensitivity of current markers remain suboptimal, often overlapping with other inflammatory conditions. Therefore, discovering new biomarkers is essential for early detection and improved therapeutic outcomes.

This study aims to computationally identify shared genomic biomarkers as potential drug targets for UC by analyzing their functions, regulatory pathways, and upstream mediators, including transcription factors and microRNAs. Additionally, candidate therapeutic compounds were evaluated through molecular docking to validate their binding affinities and interactions with key hub targets.

## 2. Materials and Methods

### 2.1. Data Collection for Gene Expression Profiles

**Figure 1:**
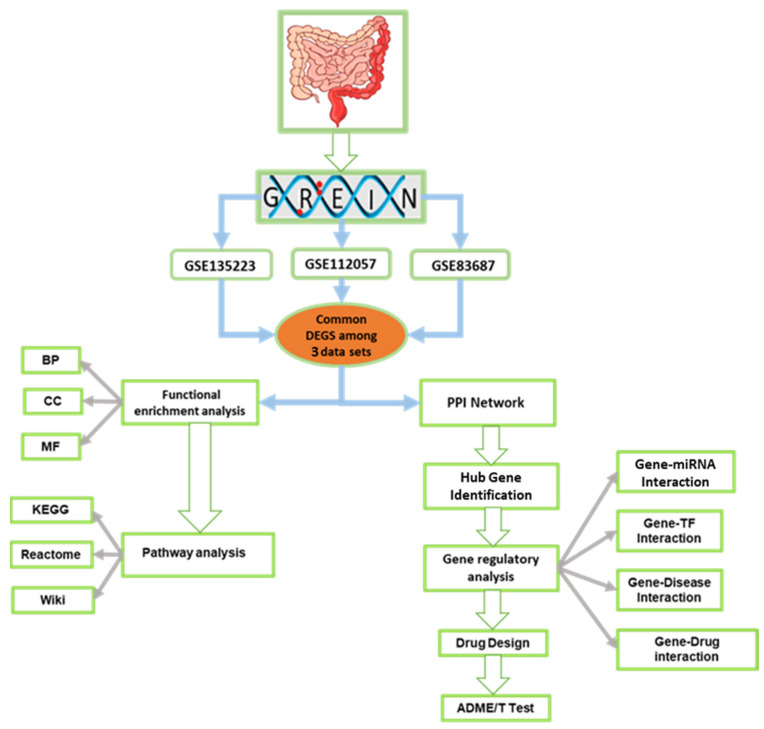
The flowchart presents a detailed schematic overview of the key steps and methodology employed in this study.

The gene expression profiles (RNA-seq data) were retrieved from the Gene Expression Omnibus (GEO) database using GREIN (http://www.ilincs.org/apps/grein/?gse, accessed on 11 October 2024) with the search term “ulcerative colitis” [17]. The selected datasets met the following inclusion criteria to ensure analytical relevance: (a) Homo sapiens; (b) mRNA expression profiling; (c) study design including both experimental (UC) and control groups; (d) a minimum of 10 samples per dataset; and (e) exclusion of cell line data. Based on these criteria, three GEO datasets-GSE135223, GSE112057, and GSE83687 were selected for comparative analysis of UC and normal tissue gene expression. A schematic overview of the study design is presented in Figure 1.

### 2.2. Screening and Identification of Differentially Expressed Genes (DEGs)

The raw expression values from each GEO dataset were preprocessed and normalized by the original submitters using the respective GEO platform pipelines, such as RMA normalization for microarray data and counts per million (CPM)/TPM normalization for RNA-seq data [18]. To ensure consistency across datasets, the expression values were log2-transformed (where applicable) and further median-centered to minimize batch effects. The normalized data were then analyzed in Excel 2016 to screen for DEGs between the UC and control groups, with genes filtered using the criteria |logFC| > 1 and *p* < 0.05. Genes with logFC > 1 were considered upregulated, while those with logFC < –1 were considered downregulated. Finally, the online tool jvenn was applied to identify DEGs common across all three datasets [19].

### 2.3. Protein–Protein Interaction (PPI) Network Analysis

The functional interactions between DEGs were assessed using a PPI network. The PPI network was constructed using the Search Tool for the Retrieval of Interacting Genes (STRING) [20]. Protein interaction data were visualized and analyzed using Cytoscape (version 3.9.1). In the PPI network, the nodes represent proteins, while the edges indicate interactions between them. In the network, nodes represent proteins and edges indicate interactions. Hub genes were identified based on their connectivity, with higher values indicating stronger centrality within the network.

### 2.4. Identification of Hub Genes

Hub genes were identified using the cytoHubba plugin in Cytoscape [21]. Twelve algorithms were applied to assess node importance, including MCC, Closeness, Radiality, Betweenness, and Stress centralities [22]. The top 10 genes from each method were selected based on scores, indicating their potential biological relevance and prioritization for further study.

### 2.5. Gene Enrichment Analysis

Functional enrichment was conducted using Database for Annotation, Visualization, and Integrated Discovery (DAVID, https://davidbioinformatics.nih.gov/tools.jsp, accessed on 23 January 2025), which provides comprehensive annotation tools [23]. Enhanced *p*-value enrichment was applied to ensure statistical significance and improve analysis accuracy. Enrichment was evaluated for biological processes, cellular components, and molecular functions. The top 10 terms in each category were selected, and the top 5 pathways were identified using KEGG, WikiPathways, and Reactome. Enrichment results were visualized using bubble plots generated via SRplot (https://www.bioinformatics.com.cn/en, accessed on 29 january 2025).

### 2.6. Gene–miRNA–TF Interaction Network

Regulatory interactions were analyzed to explore how transcription factors (TFs) and microRNAs (miRNAs) influence gene expression [24,25,26]. Gene regulatory network (GRN) analysis was performed using NetworkAnalyst (https://www.networkanalyst.ca/, accessed on 22 February 2025) and integrated data from three databases [27]. miRNA–gene interactions were sourced from miRTarBase v9.0 and TarBase v9.0, while TF–gene relationships were predicted based on ENCODE and JASPAR binding profiles.

### 2.7. Gene–Disease Association Analysis

Gene–disease associations were investigated using DisGeNET (https://www.disgenet.org/, accessed on 24 February 2025), which integrates information from multiple sources related to human diseases [28]. Network analysis helped identify diseases and long-term complications linked to shared DEGs.

### 2.8. Protein–Drug Interaction Analysis

Protein–drug interactions were analyzed using the DrugBank (version 5.0) database [29]. NetworkAnalyst was used to visualize and filter potential drug candidates targeting key DEGs for UC therapy.

### 2.9. Molecular Docking

Molecular docking was performed using AutoDock Vina via PyRx. The conjugate gradient algorithm was applied to optimize potential ligands in 200 steps using the Universal Force Field (UFF) [30]. The docking grid was centered at (x = 11.5647583557, y = −15.0323563498, z = −14.9472), with dimensions of X: 134.951379941 Å, Y: 124.914233058 Å, and Z: 63.7435282516Å. Ligands selected for further analysis exhibited higher binding affinities (kcal/mol) than the control ligand, with more negative values indicating stronger binding interactions. Only ligands with a zero root mean square deviation (RMSD) were considered. Finally, molecular interactions and structural visualizations were analyzed using Discovery Studio.

### 2.10. ADME/T Prediction

ADME/T (Absorption, Distribution, Metabolism, Excretion, and Toxicity) prediction is a crucial filtering step in computational drug design [31]. Computational methods offer a cost-effective and time-efficient alternative to labor-intensive experimental evaluations, as demonstrated in recent studies. In this study, SwissADME was employed to assess pharmacokinetic properties, including drug absorption, metabolism, distribution, and excretion [32]. Additionally, toxicity assessments were conducted using the pkCSM server [33]. Molecular structures were represented using Structure Data File (SDF) strings and the Simplified Molecular Input Line Entry System (SMILES) for accurate computational analysis.

## 3. Results

### 3.1. Identification of DEGs

**Figure 2:**
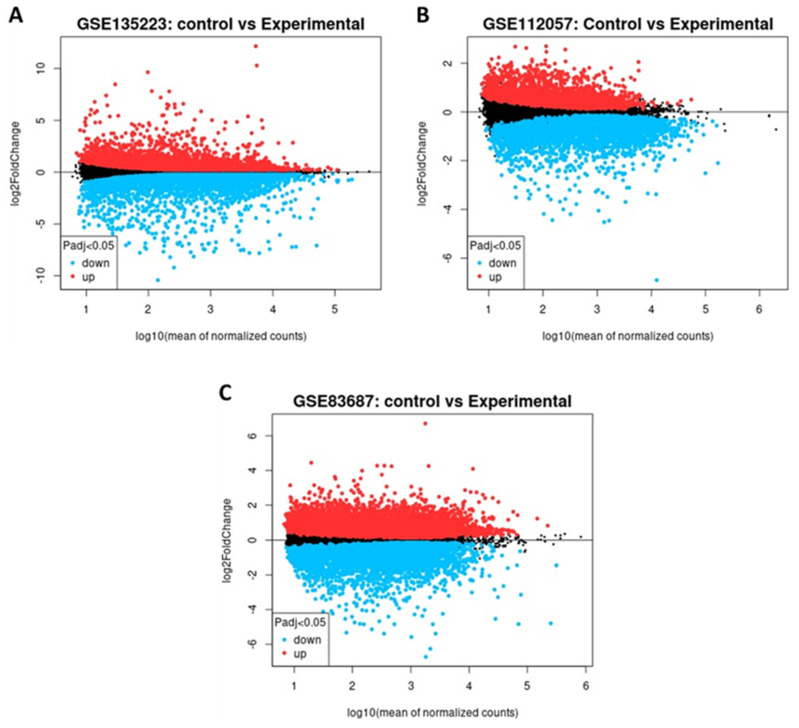
Mean–difference (MD) plots for datasets (**A**) GSE135223, (**B**) GSE112057, and (**C**) GSE83687, showing the distribution of differentially expressed genes (DEGs) between ulcerative colitis (UC) and control groups. Upregulated DEGs are represented in red, downregulated DEGs in blue, and non-significant genes in black.

A total of 2340, 1526, and 2759 significant DEGs were identified in the GSE135223, GSE112057, and GSE83687 datasets, respectively. Among these, 1,279, 905, and 1512 genes were upregulated, while 1,061, 621, and 1247 were downregulated (Table 1) and Supplementary Table S1. GSE135223 exhibited a wider range of gene expression changes, indicating notable transcriptional alterations (Figure 2A). In contrast, GSE112057 displayed a moderate distribution with a higher proportion of upregulated genes (Figure 2B), and GSE83687 showed a more compact distribution, suggesting less pronounced fold changes (Figure 2C). Most DEGs fell within the log_10_(mean expression) range of 1 to 4, indicating moderate to high expression levels.

### 3.2. Common Gene Identification and PPI Construction

**Figure 3:**
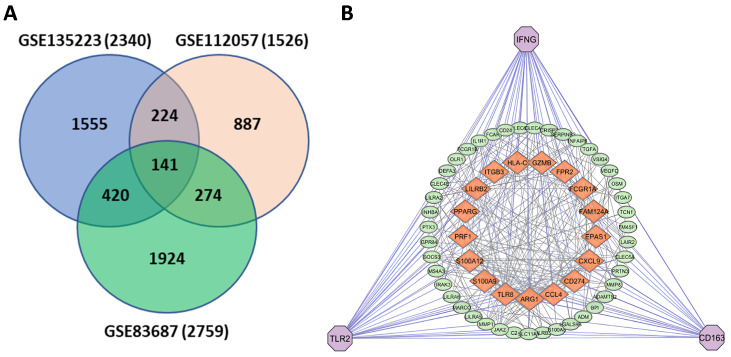
Common genes across three datasets and their protein–protein interaction (PPI) network. (**A**) Significant overlapping DEGs were identified from three selected datasets in the GEO database. (**B**) PPI network constructed using STRING at a confidence threshold of 0.7, containing 105 nodes and 366 edges. The three most highly interactive genes are represented as light pink octagons, while 17 prominently connected genes are displayed as orange squares. Key hub genes such as interferon-gamma (IFNG), toll-like receptor 2 (TLR2), and CD163 molecule (CD163) are highlighted due to their high connectivity.

The Venn diagram (Figure 3A) shows the overlap of DEGs among three datasets (GSE135223, GSE112057, and GSE83687), identifying 141 shared DEGs that may serve as key contributors to UC. The final PPI network consisted of 105 nodes and 366 edges, constructed using STRING interactions at a confidence threshold of 0.7 (Figure 3B) identifies IFNG, TLR2, and CD163 as central hub genes, with strong interactions (orange nodes) involving other significant genes such as PPARG, ARG1, CCL4, and CXCL9. These genes are closely associated with immune regulation, inflammatory signaling, and UC-related disease mechanisms, underscoring their potential as biomarkers and therapeutic targets.

### 3.3. Identification of Hub Genes

**Figure 4:**
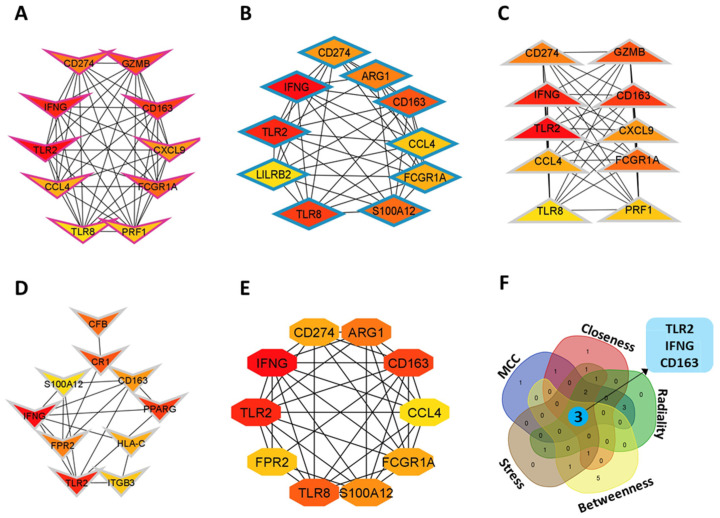
Identification of hub genes within the PPI network using cytoHubba algorithms in Cytoscape: (**A**) maximal clique centrality (MCC), (**B**) closeness, (**C**) radiality, (**D**) betweenness, and (**E**) stress centralities. Nodes are ranked by significance, with a red-to-yellow gradient indicating higher to lower scores. (**F**) Venn diagram showing three common hub genes (IFNG, TLR2, and CD163) consistently identified across all five methods.

We analyzed the PPI network derived from STRING using our gene sets and visualized it in Cytoscape to explore common adhesion pathways and DEG interactions. The network consisted of 366 edges and 105 nodes based on STRING interactions at a confidence threshold of 0.7 (Figure 3B). To ensure robustness of the network and avoid centrality bias, we evaluated hub gene stability across 12 cytoHubba ranking methods. TLR2, IFNG, and CD163 consistently ranked among the top hubs across five key centrality measures (MCC, Closeness, Radiality, Betweenness, and Stress) (Figure 4F). Varying the STRING confidence threshold between 0.4 (medium confidence) and 0.9 (high confidence) showed that these core hubs remained stable despite changes in network density. Additionally, permutation testing with 1000 randomized networks demonstrated that the observed network topology was significantly different from random (*p* < 0.001, Kolmogorov–Smirnov test), confirming the biological relevance of the identified hubs (Figure 4A–E).

### 3.4. Gene Ontology (GO) and Pathways Enrichment Analysis

The DAVID database was used to perform gene ontology and pathway enrichment analysis, helping determine the biological significance of pathways associated with DEGs. Gene ontology assesses gene functions and attributes by integrating comprehensive computational knowledge resources [34]. An ontology serves as a structured framework of biological knowledge that supports biological modeling and annotation, commonly applied in biomedical research.

**Figure 5:**
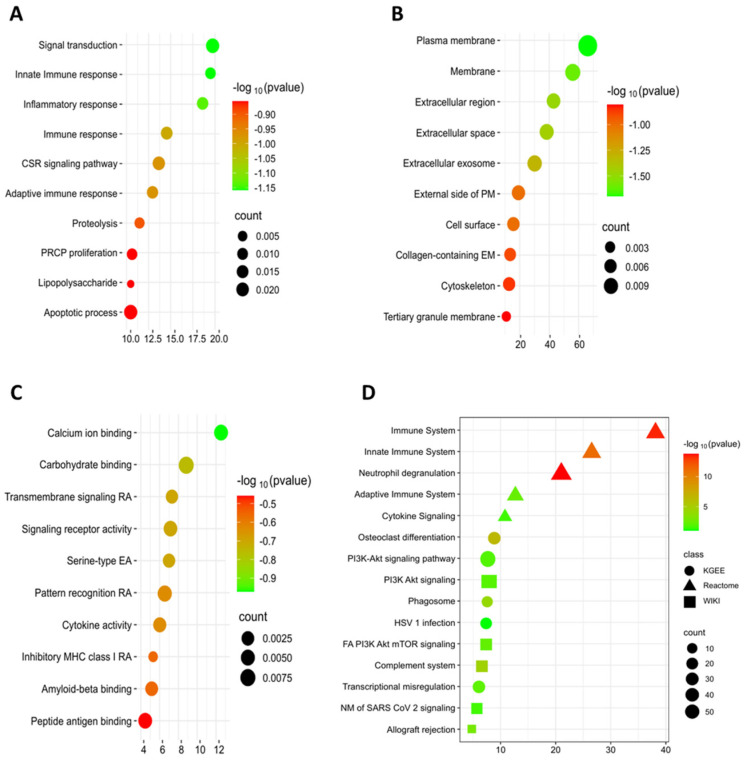
A bubble plot was generated for GO analysis based on the −log10 (*p*-value), grouping genes into (**A**) Biological Process (BP), (**B**) Cellular Components (CC), and (**C**) Molecular Function (MF). Larger bubbles indicate a higher number of genes associated with a specific process or pathway, while smaller bubbles represent fewer associated genes. Bubble colors correspond to the −log10 (*p*-value) for each term. Additionally, pathway analysis was performed, with (**D**) Pathway enrichment results integrating Kyoto Encyclopedia of Genes and Genomes (KEGG, circles), Reactome (triangles), and WikiPathways (squares)., following the same size and color significance criteria.

For GO analysis, three major domains were considered: molecular function, cellular component, and biological process [35]. The most frequently enriched GO terms included signal transduction, inflammatory response, membrane, extracellular region, carbohydrate binding, and transmembrane activity. Bubble plots illustrate the enrichment results across each category (Figure 5A–C). Additionally, several significant molecular pathways were identified, including the phagosome pathway and PI3K-Akt signaling pathway (KEGG), the innate immune system pathway (Reactome), and the focal adhesion PI3K-Akt-mTOR signaling pathway (WikiPathways), as shown in Figure 5D. Full GO annotation details and pathway enrichment results are provided in Table 2 and Table 3. Also, Full data is available in Supplementary Tables S2 and S3.

### 3.5. Identification of Gene Regulators

**Figure 6:**
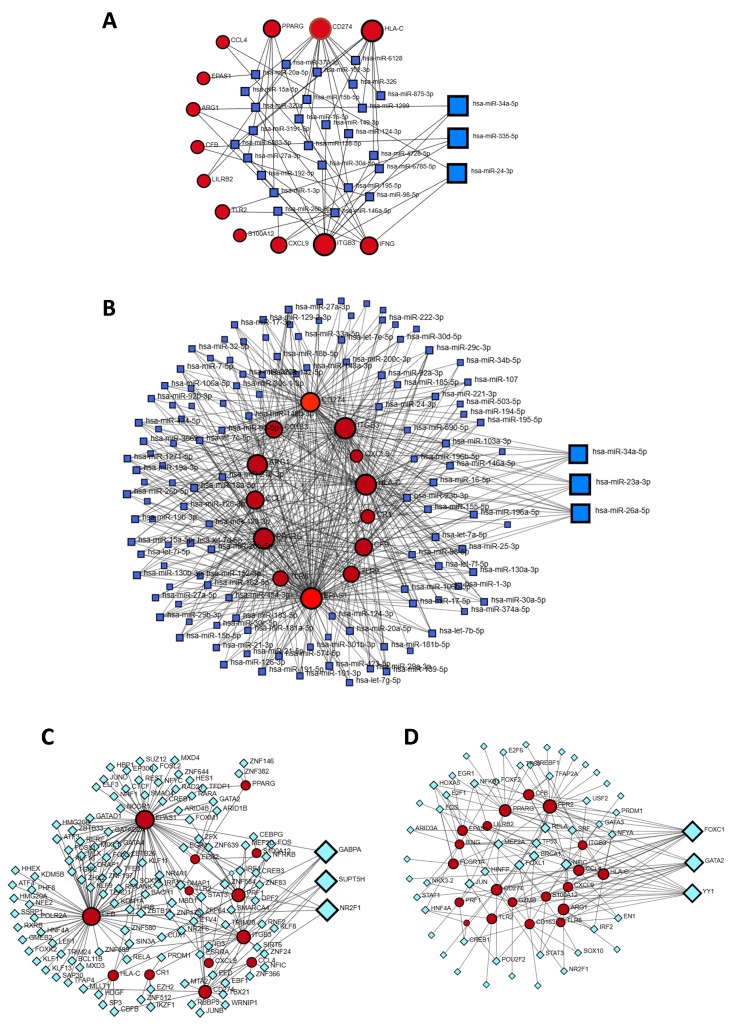
Regulatory interaction networks of DEGs with microRNAs (miRNAs) and transcription factors (TFs). (**A**,**B**) DEG–miRNA networks constructed using miRTarBase v9.0 and TarBase v9.0. miRNAs are shown as squares, and DEGs as circles. (**C**,**D**) TF–DEG networks based on ENCODE and JASPAR databases, with TFs represented as diamonds and DEGs as circles.

Gene–miRNA interactions were analyzed using the NetworkAnalyst platform, incorporating data from the miRTarBase v9.0 database. This revealed 42 miRNAs linked to 13 seed genes through 61 edges (betweenness = 20), with hsa-miR-34a-5p, hsa-miR-335-5p, and hsa-miR-24-3p emerging as the top regulators. Analysis using the TarBase v9.0 database identified 306 miRNAs associated with the same 13 genes via 509 edges, with hsa-miR-34a-5p, hsa-miR-23a-5p, and hsa-miR-26a-5p showing the strongest influence. Notably, hsa-miR-34a-5p was common to both datasets (Figure 6A,B).

For transcription factor (TF) analysis, ENCODE and JASPAR databases were used. ENCODE identified 142 TFs interacting with 13 genes across 216 edges (betweenness = 40), with GABPA, SUPT5H, and NR2F1 being the most prominent. JASPAR revealed 77 TFs linked to 20 genes via 172 edges (betweenness = 10), highlighting FOXC1, GATA2, and YY1 as key regulators within the TF-DEG network (Figure 6C,D).

### 3.6. Identification of Disease Associations and Drug Candidates

**Figure 7:**
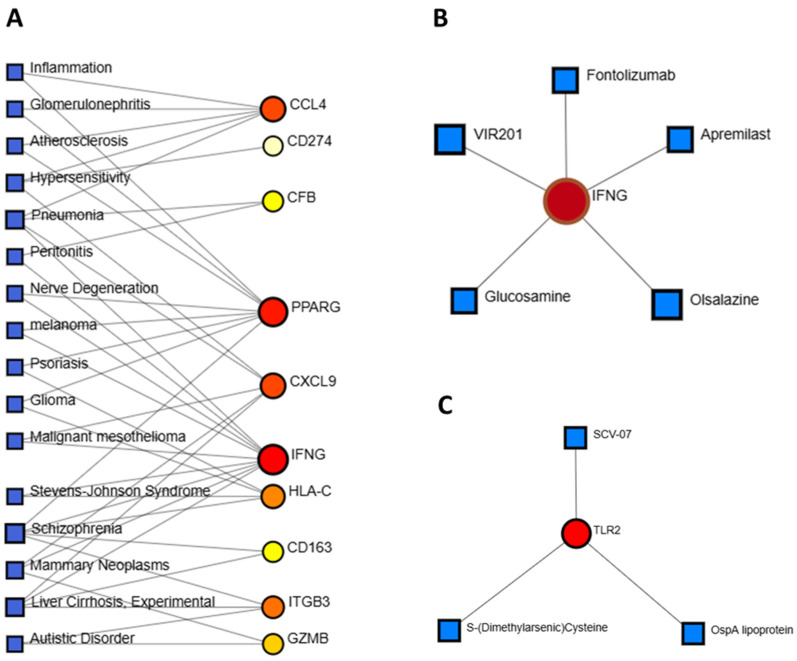
(**A**) Gene–disease association network constructed using DisGeNET, showing links between hub genes (red circles) and related diseases (blue squares). (**B**,**C**) Gene–drug interaction networks based on DrugBank data. Red circles represent hub gene targets, while blue squares represent candidate pharmaceutical compounds.

To explore gene–disease associations, we used NetworkAnalyst with integrated DisGeNET data. Ten genes, IFNG, CD163, CCL4, CFB, CXCL9, HLA-C, ITGB3, GZMB, CD274, and PPARG, were frequently associated with five disorders: schizophrenia, hypersensitivity, pneumonia, mammary neoplasms, and liver cirrhosis (Figure 7A). Protein–drug interaction analysis identified potential UC therapeutics. Five drugs, Fontolizumab, Apremilast, Olsalazine, Glucosamine, and VIR201 were found to target IFNG (Figure 7B). Three additional compounds, SCV-07 (Golotimod), OspA lipoprotein, and S-(Dimethylarsenic)-cysteine targeted TLR2 (Figure 7C).

### 3.7. Molecular Docking for Drug Repurposing and ADME/T Profiling

**Figure 8:**
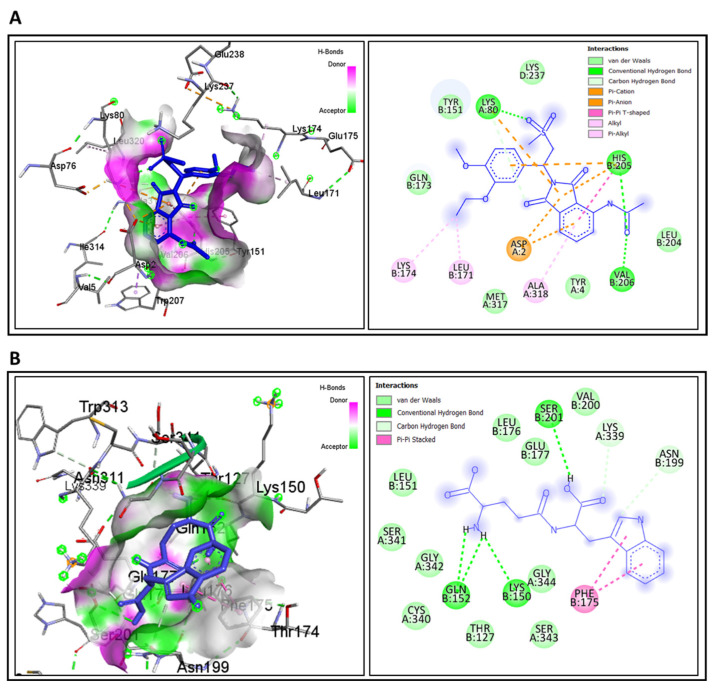
Molecular docking interactions of candidate therapeutic agents with hub protein targets. (**A**) Binding interactions of Apremilast with interferon-gamma (IFNG). (**B**) Binding interactions of Golotimod with toll-like receptor 2 (TLR2). Hydrogen bonds and hydrophobic interactions are illustrated.

To identify potential therapeutic candidates for UC, molecular docking was performed to assess binding affinities between FDA-approved compounds and hub protein targets. The final hub-proteins were selected as drug target receptors, and candidate ligands from DrugBank were screened for docking analysis. Among the two receptor proteins and eight candidate drugs tested, the top two lead compounds-Apremilast (CID: 11561674) and Golotimod (CID: 6992140) exhibited the most significant binding affinities, measured at −8.3 kcal/mol and −7.3 kcal/mol, respectively. The corresponding binding amino acid residues are detailed in Table 4, with molecular docking interactions visualized in Figure 8.

The ADME/T analysis compares the physicochemical, pharmacokinetic, and toxicological properties of Apremilast, Golotimod, Mesalamine, and Azathioprine. All compounds meet Lipinski’s rule, indicating favorable drug-likeness. Apremilast had the highest molecular weight (460.50 g/mol), whereas Mesalamine is the smallest (153.14 g/mol). Apremilast also demonstrates the highest intestinal absorption (83.79%), while Golotimod exhibits lower absorption (56%). None of the compounds significantly crossed the blood–brain barrier (BBB). Apremilast functioned as a CYP3A4 substrate/inhibitor, potentially influencing drug metabolism; Golotimod exhibited the highest total clearance (0.595 mL/min/kg), while Azathioprine had the lowest (0.148 mL/min/kg). The toxicity analysis revealed AMES toxicity for Mesalamine and Azathioprine, but not for Apremilast or Golotimod (Table 5).

## 4. Discussion

Genome-wide association studies (GWAS) have greatly advanced the understanding of polygenic disorders, particularly in identifying several genes associated with UC. These findings have provided key insights into the disease’s pathophysiology, which involves a complex interaction between genetic and environmental factors, leading to diverse clinical outcomes. Nonetheless, the exact etiology of UC remains incompletely understood and highly complex.

In this study, we identified 2340, 1526, and 2759 significant DEGs in datasets GSE135223, GSE112057, and GSE83687, respectively. Among them, 1279, 905, and 1512 were upregulated, and 1061, 621, and 1247 were downregulated. Gene Ontology functional classification revealed enrichment in pathways related to signal transduction, inflammatory response, membrane, extracellular region, carbohydrate binding, and transmembrane activity. Chronic inflammation in inflammatory bowel disease (IBD) may arise from microbial infections or impaired mucosal barrier function, compounding disease progression.

A PPI network analysis of DEGs identified 20 key genes- including GZMB, FPR2, FCGR1A, FAM124A, EPAS1, CXCL9, CD274, CCL4, ARG1, TLR8, S100A9, S100A12, PRF1, PPARG, LILRB2, ITGB3, HLA-C, TLR2, IFNG, and CD163, among 141 genes with the highest interaction levels. Previous studies have demonstrated that these genes play a crucial role in the development and progression of UC and its related complications [36,37,38,39,40,41,42,43,44]. Among these 20 genes, our analysis focused on identifying the most promising biomarkers, with TLR2, IFNG, and CD163 emerging as pivotal in UC pathogenesis due to their involvement in immune response modulation and inflammation. The studies by Medzhitov (2001) and Vasselon & Detmers (2002) established that TLR2 (Toll-like receptor 2) plays a critical role in recognizing microbial components and initiating innate immune responses [45,46]. Dysregulation of TLR2 has been linked to the excessive activation of NF-κB signaling, resulting in an upsurge of pro-inflammatory cytokines that exacerbate mucosal inflammation in UC patients, as previously described by Tatiya-Aphiradee et al. (2018) [47]. Similarly, IFNG (Interferon-γ), a cytokine predominantly produced by T-cells and natural-killer (NK) cells, is integral to macrophage activation and antigen presentation [48]. Elevated IFNG expression levels have been associated with chronic inflammation and tissue damage in UC, leading to increased immune cell infiltration and cytokine imbalance, as reported by Tatiya-Aphiradee et al. (2018) [47]. Additionally, CD163, a scavenger receptor expressed on macrophages, plays a significant role in hemoglobin-haptoglobin complex clearance and anti-inflammatory modulation [49]. Recent findings by Zhang et al. (2025) indicate that CD163 expression in UC is often upregulated as a compensatory mechanism to counteract inflammation, but its overexpression may also contribute to fibrosis and impaired tissue repair [50]. Understanding the interplay between these genes provides essential insights into UC pathogenesis while offering potential therapeutic targets for future interventions. Furthermore, these three genes-TLR2, IFNG, and CD163-are abundantly expressed in colorectal cancer (CRC) tumor tissues. Previous studies suggest that both spontaneous and inflammation-driven CRC proliferation can be mitigated through gene knockdown and genetic deletion.

In Gene Ontology analysis, key biological processes such as signal transduction, innate immune response, and inflammatory response were identified as significant contributors to UC development and progression. Major cellular components, including the plasma membrane, extracellular region, and exosomes, play crucial roles in cellular communication, facilitating the transport of proteins, miRNAs, lncRNAs, and circRNAs from UC-affected to normal cells [51]. Exosomes influence proliferation, adhesion, invasion, and angiogenesis, thereby promoting UC tumor progression [52]. Moreover, a study by Liang et al. (2024) revealed that calcium ion binding activity is a critical molecular function, potentially serving as an independent risk factor for UC [53].

Pathway analysis uncovered key signaling mechanisms implicated in UC progression. Within the KEGG, processes such as immune system regulation, neutrophil degranulation, and cytokine signaling were identified as pivotal. Reactome pathway analysis highlighted osteoclast differentiation and the PI3K-Akt signaling pathway as major contributors, while WikiPathway emphasized the FA PI3K-Akt-mTOR signaling pathway and the complement system, both playing significant roles in UC development. Previously studies have demonstrated that these pathways collectively influence inflammation, immune response, and cellular survival, underscoring their relevance in UC pathogenesis [54,55,56].

Using NetworkAnalyst, we identified five key miRNAs with significant roles in UC and other diseases, including hsa-miR-34a-5p, hsa-miR-335-5p, hsa-miR-24-3p, hsa-miR-23a-5p, and hsa-miR-26a-5p, sourced from miRTarBase and TarBase databases. Notably, hsa-miR-34a-5p was found in both databases, indicating its central role in UC pathogenesis. Studies by Ojha et al. (2019) and Blauensteiner & Westermeier (2021) revealed that hsa-miR-34a-5p regulates inflammatory pathways, immune responses, and epithelial barrier integrity [57,58]. Its dysregulation enhances NF-κB signaling, cytokine production, and gut permeability by downregulating tight junction proteins, contributing to chronic inflammation and mucosal damage, making it a potential biomarker and therapeutic target [57]. Similarly, hsa-miR-335-5p, hsa-miR-24-3p, hsa-miR-23a-5p, and hsa-miR-26a-5p exhibit critical functions in UC, modulating inflammation, immune responses, and epithelial integrity. Sun & Huang (2019) demonstrated that hsa-miR-335-5p modulates cytokine expression [59], while He et al. (2023) presented evidence that hsa-miR-24-3p influences cell proliferation and apoptosis [60]. Additionally, studies by Marchese et al. (2018) and Li et al. (2024) identified hsa-miR-23a-5p as a key factor in T-cell activation and hsa-miR-26a-5p as essential for maintaining intestinal barrier function [61,62]. Their dysregulation may contribute to UC progression, highlighting their potential as biomarkers or therapeutic targets.

In this study, we identified FOXC1, GABPA, GATA2, and SUPT5H as key transcription factors implicated in the development of ulcerative colitis (UC) through the regulation of gene expression involved in inflammation, immune response, and epithelial barrier integrity. FOXC1 (Forkhead Box C1) plays a critical role in immune cell differentiation and inflammation, contributing to mucosal damage in UC [63]. GABPA (GA Binding Protein Transcription Factor Alpha Subunit) is integral to mitochondrial biogenesis and immune regulation, ensuring cellular homeostasis in inflamed colonic tissues [64]. GATA2 (GATA Binding Protein 2) is essential for hematopoiesis and immune cell function, impacting cytokine production and immune responses in UC [65]. SUPT5H, a transcription elongation factor, regulates RNA polymerase II activity and may modulate the expression of inflammatory genes [66]. The dysregulation of these TFs can result in impaired epithelial repair, increased inflammation, and disrupted gut homeostasis, making them potential biomarkers and therapeutic targets in UC management.

Additionally, we identified two potential drugs capable of binding to hub proteins and regulating their activity effectively. Apremilast (CID: 11561674) and Golotimod (CID: 6992140) emerged as promising therapeutic agents for UC treatment, targeting key inflammatory pathways. Apremilast, a PDE4 inhibitor, modulates cAMP levels, downregulating pro-inflammatory cytokines like IFNG (Interferon-γ) to reduce immune activation, prevent tissue damage, and promoting mucosal healing [67,68]. Meanwhile, Golotimod, an immunomodulatory peptide, inhibits TLR2 signaling, curbing excessive cytokine production and enhancing epithelial barrier function [69]. Both drugs exhibit strong potential for inflammation control and UC remission maintenance, warranting further clinical investigation. A comparative analysis of pharmacokinetic and physicochemical properties supports Apremilast and Golotimod as promising candidates for UC treatment. Both compounds conform to Lipinski’s rule, signifying good drug-likeness without violations. Unlike control drugs Mesalamine and Azathioprine, which demonstarted AMES toxicity, Apremilast and Golotimod remain non-mutagenic, reinforcing their potential as safer and more effective therapeutic options for UC management.

Previous studies have identified several UC biomarkers, including HNF4A, ICAM1, and RNF186 for epithelial barrier integrity [14,16], as well as calprotectin, CRP, and OSM for diagnosis and treatment response [39,41]. In contrast, our study highlights TLR2, IFNG, and CD163 as key hub genes with immune-regulatory roles. Notably, CD163 links macrophage polarization and fibrosis to UC pathology [49,50], providing a novel insight. Furthermore, drug–target analysis suggests that compounds such as Apremilast and Golotimod may modulate these pathways, indicating potential for therapeutic repurposing [47,48] (Table 6).

**Table 6:** Comparison of validated ulcerative colitis (UC) biomarkers with hub genes identified in this study.

Category	Validated UC/Treatment Markers (Previous Studies)	Functional Role in UC	Hub Genes Identified in This Study	Functional Role in UC (This Study)
**Diagnostic/Disease Activity**	Calprotectin (S100A8/A9) [41], C-reactive protein (CRP) [41]	Clinical biomarkers widely used for inflammation and disease monitoring	CD163	Scavenger receptor on macrophages; associated with anti-inflammatory modulation, fibrosis, and impaired tissue repair [49,50]
**Genetic Predisposition/Barrier Integrity**	HNF4A, ICAM1, RNF186 [14,16]	Regulate epithelial barrier integrity, immune modulation, and genetic susceptibility to UC	TLR2	Recognizes microbial ligands; initiates innate immune responses; dysregulation activates NF-κB and exacerbates mucosal inflammation [45,46,47]
**Inflammation/Immune Signaling**	Oncostatin M (OSM) [39], TNF-α, IL-6 [47]	Pro-inflammatory mediators; OSM predicts anti-TNF therapy resistance	IFNG	Key cytokine driving macrophage activation, T-cell infiltration, and chronic inflammation in UC [47,48]
**Therapeutic Implications**	Anti-TNF response markers (e.g., OSM, CRP) [39,41]	Guide biologic therapy decisions	Apremilast, Golotimod (predicted drug–gene interactions)	Target IFNG and TLR2; potential drug repurposing candidates with favorable ADME/T properties [67,68,69]

This study is entirely based on secondary bioinformatics analyses of publicly available transcriptomic datasets. As such, it lacks experimental validation in cell-based or animal models, and the identified biomarkers and drug candidates require further confirmation in clinical cohorts. Additionally, dataset heterogeneity and sample size variations may introduce bias, limiting generalizability. Despite these limitations, the findings provide clinically relevant insights. The identification of TLR2, IFNG, and CD163 as hub genes suggests their potential utility as predictive biomarkers for ulcerative colitis diagnosis and disease monitoring. Moreover, the computational prediction of Apremilast and Golotimod as promising therapeutic agents highlights a possible drug repurposing strategy that could complement current therapies, reduce treatment resistance, and support personalized medicine approaches in UC management.

## 5. Conclusions

This study identified key hub genes (TLR2, IFNG, CD163), transcription factors (FOXC1, GABPA, GATA2, SUPT5H), and microRNAs (hsa-miR-34a-5p, hsa-miR-335-5p, hsa-miR-24-3p, hsa-miR-23a-5p, hsa-miR-26a-5p) that regulate ulcerative colitis progression through immune regulation and PI3K-Akt-mTOR signaling pathways. Molecular docking predicted Apremilast and Golotimod as promising therapeutic candidates, with favorable pharmacokinetic and safety profiles compared to conventional drugs. Although limited by its in silico design and lack of experimental validation, this study provides insights into potential biomarkers and therapeutic targets that may guide personalized diagnosis and treatment strategies in UC, warranting further preclinical and clinical validation.

## Figures and Tables

**Table 1:** Summary of the differentially expressed genes (DEGs) identified in selected mRNA expression datasets, comparing UC and control cohorts across various cell types.

GEO Accession No	Source of Sample	Total Sample Size	Control Sample	Case Sample	Experiment Type	Total DEGs	Up-Regulated DEGs	Down- Regulated DEGs
GSE135223	Colon, Macrophages	10	5	5	Stranded RNA sequencing	2340	1279	1061
GSE112057	Colon, Macrophages, small bowel	27	12	15	Stranded RNA sequencing	1526	905	621
GSE83687	Colon, rectum, small bowel	92	60	32	Stranded RNA sequencing	2759	1512	1247

**Table 2:** Primary UC-related genes were associated with the top five GO terms in each of the three major categories, totaling 15 key terms.

Biological Process						
Pathway Name	ID	*p*-Value	Enrichment	Number of Genes Involved	Benjamini	FDR < 0.05
Innate Immune response	GO:0045087	4.73 × 10^−09^	14.38849	20	2.43 × 10^−06^	2.37 × 10^−06^
Signal transduction	GO:0007165	9.97 × 10^−04^	14.38849	20	0.040968	0.040051
Inflammatory response	GO:0006954	3.28 × 10^−10^	13.66906	19	3.37 × 10^−07^	3.29 × 10^−07^
Immune response	GO:0006955	4.41 × 10^−05^	10.07194	14	0.005413	0.005292
CSR signaling pathway	GO:0007166	3.37 × 10^−06^	9.352518	13	8.94 × 10^−04^	8.74 × 10^−04^
**Cellular component**						
Plasma membrane	GO:0005886	4.14 × 10^−10^	50.35971	70	4.12 × 10^−08^	3.85 × 10^−08^
Membrane	GO:0016020	1.06 × 10^−04^	40.28777	56	0.002001	0.00187
Extracellular region	GO:0005576	2.49 × 10^−10^	30.21583	42	4.12 × 10^−08^	3.85 × 10^−08^
Extracellular space	GO:0005615	1.41 × 10^−09^	27.33813	38	9.36 × 10^−08^	8.75 × 10^−08^
Extracellular exosome	GO:0070062	8.55 × 10^−04^	20.14388	28	0.011349	0.010608
**Molecular Function**						
Calcium ion binding	GO:0005509	0.004742	9.352518	13	0.13474	0.131579
Carbohydrate binding	GO:0030246	5.12 × 10^−04^	5.755396	8	0.058147	0.056782
Transmembrane signaling RA	GO:0004888	0.001923	5.035971	7	0.098729	0.096412
Serine-type EA	GO:0004252	0.002027	5.035971	7	0.098729	0.096412
Signaling receptor activity	GO:0038023	0.004322	5.035971	7	0.13474	0.131579

**Table 3:** Key UC-related targets were associated with the top five enriched signaling pathways in each of the three major categories.

KEGG						
Pathway Name	ID	*p*-Value	Enrichment	Number of Genes Involved	Benjamini	FDR < 0.05
Osteoclast differentiation	hsa04380	3.80 × 10^−07^	7.913669	11	6.80 × 10^−05^	6.34 × 10^−05^
Phagosome	hsa04145	6.62 × 10^−05^	6.47482	9	0.0059204	0.005524
PI3K-Akt signaling pathway	hsa04151	0.012915	6.47482	9	0.151348233	0.141202
Herpes simplex virus 1 infection	hsa05168	0.082924	6.47482	9	0.549757891	0.512903
Transcriptional mis regulation in cancer	hsa05202	0.007328	5.035971	7	0.1293794	0.120706
**Reactome Pathways**						
Immune System	R-HSA-168256	3.94 × 10^−14^	38.84892	54	9.47 × 10^−12^	9.35 × 10^−12^
Innate Immune System	R-HSA-168249	8.31 × 10^−12^	25.89928	36	1.33 × 10^−09^	1.32 × 10^−09^
Neutrophil degranulation	R-HSA-6798695	1.80 × 10^−14^	20.14388	28	8.66 × 10^−12^	8.55 × 10^−12^
Adaptive Immune System	R-HSA-1280218	0.004189	11.51079	16	0.201481011	0.198968
Cytokine Signaling in Immune system	R-HSA-1280215	0.027725	10.07194	14	0.514440567	0.508023
**WikiPathways**						
Focal adhesion PI3K Akt mTOR signaling	WP3932	0.005393	6.47482	13	0.190108	0.186063
PI3K Akt signaling	WP4172	0.01035	6.47482	13	0.291878	0.285668
Complement system	WP2806	2.01 × 10^−05^	5.755396	12	0.005662	0.005541
Network map of SARS CoV 2 signaling	WP5115	0.025447	5.035971	12	0.598015	0.585291
Allograft rejection	WP2328	0.001205	4.316547	8	0.068002	0.066555

**Table 4:** Binding interactions between top two lead compounds and two control lead compounds with the top two potential target proteins based on AutoDock Vina results.

Name of Potential Targets (PDB ID)	“Name” and Compound ID of Drugs (2 Control Drugs)	Docking Score of Controls (Kcal/mol)	Docking Score of Drugs (Kcal/mol)	Amino Acids Interaction of Drugs		Number of Total Bonds
				Hydrogen Bond	Hydrophobic Bond	
**IFNG** **(IFYH)**	“Apremilast” CID: 11561674 Mesalamine CID: 4075 Azathioprine CID: 2265	−5.2 −5.8	−8.3	LYS A: 80, LYS D: 237, TYR B: 151, HIS B: 205, VAL B: 206, LEU B: 204, TYR A: 4, MET A:317, GLN B:173	LYS B: 174, LEU B: 171, ALA A: 318, ASP A: 2	13
**TLR2** **(2Z80)**	“Golotimod” CID:6992140 (Mesalamine CID: 4075) (Azathioprine CID: 2265)	−5.9 −6.7	−7.6	VAL B: 200, SER B: 201, GLN B: 152, LYS B:150, LEU B: 176, GLU B: 177, LEU B: 151, SER A: 341, GLY A:342, CYS A:340, THR B:127, GLY A:344. SER A: 343	LYS A: 339, ASN B: 199, PHE B: 175	16

**Table 5:** Comparative pharmacokinetic profiles and toxicity assessments of two candidates and two control compounds.

Properties		Compound:01 CID:11561674 (Apremilast)	Compound:02 CID:6992140 (Golotimod)	Control: 01 CID: 4075 (Mesalamine)	Control:2 CID:2265 (Azathioprine)
**Physicochemical Properties**	MW (g/mol)	460.50	333.34	153.14	277.26
	Heavy atoms	32	24	11	19
	Rotatable bonds	9	9	6	14
	H-bond acceptors	7	6	3	6
	H-bond donors	1	5	3	1
	Bioavailability Score	0.55	0.56	0.56	0.55
	PAINS	no alert	no alert	No alert	No alert
**Drug** **likeness**	Lipinski	Yes; no violation	Yes; no violation	Yes; no violation	Yes; no violation
**Absorption**	Water solubility ((log mol/L))	−4.462	−2.892	−2.109	−2.891
	Caco2 permeability (log Papp in 10^−6^ cm/s)	0.205	−0.662	0.601	0.616
	Intestinal absorption (% Absorbed)	83.793	56.004	79.024	78.64
	Skin Permeability (log Kp)	−2.761	−2.735	−2.735	−2.735
**Distribution**	VDss (log L/kg)	−0.386	−0.05	−1.629	0.138
	BBB permeability (log BB)	−0.573	−1.028	−0.6	−1.227
	CNS permeability (log PS)	−2.44	−3.496	−3.306	−3.509
**Metabolism**	CYP2D6 substrate	No	No	No	No
	CYP3A4 substrate	Yes	No	No	No
	CYP2D6 inhibitor	No	No	No	No
	CYP3A4 inhibitor	Yes	No	No	No
**Excretion**	Total Clearance	0.227	0.595	0.49	0.148
	Renal OCT2 substrate	No	No	No	No
**Toxicity**	AMES toxicity	No	No	Yes	Yes
	Max. tolerated dose (log mg/kg/day)	0.042	0.738	1.153	0.491
	Skin Sensitization	No	No	No	No
	Oral Rat Acute Toxicity (LD50) (mol/kg)	2	2.477	1.809	2.486

## Data Availability

The data presented in this study using the GSE135223, GSE112057, and GSE83687 datasets are publicly available in the NCBI Gene Expression Omnibus database (GEO, http://www.ncbi.nlm.nih.gov/geo, accessed on 11 October 2024).

## Supplementary Materials

The following supporting information can be downloaded at: https://www.mdpi.com/article/10.3390/genes16111296/s1, Table S1: List of DEGs identified in the GSE135223, GSE112057, and GSE83687 datasets; Table S2: List of GO annotations; Table S3: List of identified Pathways.

## Abbreviations

The following abbreviations are used in this manuscript:

UC	Ulcerative colitis
GO	Gene ontology
KEGG	Kyoto Encyclopedia of Genes and Genomes
BP	Biological process
CC	Cellular component
MF	Molecular function
DEG	Differentially expressed gene
ENCODE	Encyclopedia of DNA Elements
TF	Transcription factors
miRNA	micro RNA
IFNG	Interferon-gamma
TLR2	Toll-like receptor 2
FOXC1	Forkhead Box C1
GABPA	GA-binding protein alpha chain
GATA2	GATA Binding Protein 2
SUPT5H	Transcription elongation factor SPT5
GRN	Gene regulatory network
